# Current and future climate- and air pollution-mediated impacts on human health

**DOI:** 10.1186/1476-069X-8-S1-S8

**Published:** 2009-12-21

**Authors:** Ruth M Doherty, Mathew R Heal, Paul Wilkinson, Sam Pattenden, Massimo Vieno, Ben Armstrong, Richard Atkinson, Zaid Chalabi, Sari Kovats, Ai Milojevic, David S Stevenson

**Affiliations:** 1 School of GeoSciences, King's Buildings, University of Edinburgh, West Mains Road, Edinburgh, UK; 2 School of Chemistry, King's Buildings, University of Edinburgh, West Mains Road, Edinburgh, UK; 3 PERHU, London School of Hygiene & Tropical Medicine, Keppel Street, London, UK; 4 Division of Community Health Sciences, St. George's, University of London, London, UK

## Abstract

**Background:**

We describe a project to quantify the burden of heat and ozone on mortality in the UK, both for the present-day and under future emission scenarios.

**Methods:**

Mortality burdens attributable to heat and ozone exposure are estimated by combination of climate-chemistry modelling and epidemiological risk assessment. Weather forecasting models (WRF) are used to simulate the driving meteorology for the EMEP4UK chemistry transport model at 5 km by 5 km horizontal resolution across the UK; the coupled WRF-EMEP4UK model is used to simulate daily surface temperature and ozone concentrations for the years 2003, 2005 and 2006, and for future emission scenarios. The outputs of these models are combined with evidence on the ozone-mortality and heat-mortality relationships derived from epidemiological analyses (time series regressions) of daily mortality in 15 UK conurbations, 1993-2003, to quantify present-day health burdens.

**Results:**

During the August 2003 heatwave period, elevated ozone concentrations > 200 μg m^-3 ^were measured at sites in London and elsewhere. This and other ozone photochemical episodes cause breaches of the UK air quality objective for ozone. Simulations performed with WRF-EMEP4UK reproduce the August 2003 heatwave temperatures and ozone concentrations. There remains day-to-day variability in the high ozone concentrations during the heatwave period, which on some days may be explained by ozone import from the European continent.

Preliminary calculations using extended time series of spatially-resolved WRF-EMEP4UK model output suggest that in the summers (May to September) of 2003, 2005 & 2006 over 6000 deaths were attributable to ozone and around 5000 to heat in England and Wales. The regional variation in these deaths appears greater for heat-related than for ozone-related burdens.

Changes in UK health burdens due to a range of future emission scenarios will be quantified. These future emissions scenarios span a range of possible futures from assuming current air quality legislation is fully implemented, to a more optimistic case with maximum feasible reductions, through to a more pessimistic case with continued strong economic growth and minimal implementation of air quality legislation.

**Conclusion:**

Elevated surface ozone concentrations during the 2003 heatwave period led to exceedences of the current UK air quality objective standards. A coupled climate-chemistry model is able to reproduce these temperature and ozone extremes. By combining model simulations of surface temperature and ozone with ozone-heat-mortality relationships derived from an epidemiological regression model, we estimate present-day and future health burdens across the UK. Future air quality legislation may need to consider the risk of increases in future heatwaves.

## Background

Air pollution is the environmental factor with the greatest impact on health in Europe [1]. Tropospheric ozone is a major contributor to this health burden [2-4]. It is a trans-boundary pollutant, whose concentrations are affected not only by regional emissions of precursor species but also by long-range transport of air from upwind continents and the stratosphere. Despite regional legislative controls on emissions of ozone precursors, background ozone levels in Europe have increased [5] due to a rise in the global ozone background concentrations probably partly related to population growth and rapid industrialization in developing nations. Future greenhouse-gas induced climate warming and emissions of ozone precursors have the potential to adversely impact human health through enhanced heat and air pollution related mortalities. Change in climate will also increase the frequency and intensity of extreme climate events such as heat waves [6]. Because ozone is generated by photochemical processes, its levels can rise substantially on hot sunny days, particularly when these are associated with slow-moving anticyclonic weather systems and stagnant air that traps emissions in the boundary layer. Hence, the direct health impacts of high temperatures can potentially be exacerbated due to high ozone levels.

One estimate suggests that 11/*28*% (with/*without *assumption of an O_3 _health threshold of 100 μg m^-3^) of the 2,045 deaths in England and Wales attributed to the heat wave of August 2003 may have been related to ozone [7]. A more detailed analysis of nine French cities in the 2003 heat wave showed appreciable variation in the contribution of ozone and heat to mortality, suggesting the influence of environmental and population modifiers [8]. One modelling study suggests that the temperatures experienced during the 2003 heatwave will become the average for summers from 2060 onwards [9].

Numerous studies have examined the effect of extreme temperatures or heatwaves on mortality rates controlling for air pollutants and epidemics [10,11], or have investigated mortality associated with ozone exposure [12,13]. However, the literature on the synergism between heat and ozone effects on human health is sparse [8,14]. Furthermore, studies to date have generally been restricted to individual cities with limited spatial variation, since the majority of fixed-site monitors that provide temperature and ozone measurements are based in urban locations.

In our NERC-funded project we begin to address this issue by: (i) quantifying the interaction between ozone-heat and mortality across 15 UK conurbations; (ii) producing high resolution projections of present-day and future temperature and ozone concentrations over the UK; and (iii) combining results from (i) and (ii) to derive estimates of present-day and future health burdens for the entire UK. This approach permits the inclusion of rural locations, in which ozone concentrations are generally higher as compared to urban locations, in our analyses, as well as allow us to examine the full range of spatial heterogeneity from urban to rural areas across the UK for present-day. Furthermore, only a few studies have used climate and chemistry transport models to assess future impacts of climate or emission scenarios on human health [13,15], and no study has yet been performed for the UK at fine-scale resolution.

## Project overview

Our overall project aim is to combine statistical epidemiological analyses of present-day mortality-ozone-temperature relationships based on data from 15 conurbations in England and Wales, with climate-chemistry model simulations to quantify the temperature-ozone mortality relationships across the entire UK at 5 km by 5 km horizontal resolution for present-day and under a range of future emission scenarios across the UK. The emissions scenarios span a range of possible emission futures from optimistic ("Maximum feasible reduction"), pessimistic (IPCC SRESA2) and current legislation [16]. Present-day and future health burdens will be estimated and these will be evaluated within the context of adaptation and mitigation options.

## Methods

### Environmental data

**Figure 1:**
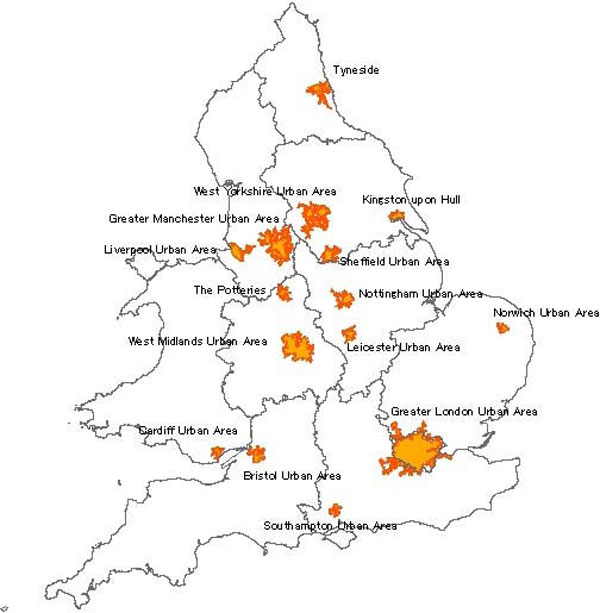
**Map indicating the 15 UK conurbations for detailed focus in this study**.

Data from urban background air quality monitoring stations (obtained from the UK Air Quality Archive, http://www.airquality.co.uk) and meteorological weather stations (obtained from the British Atmospheric Data Centre, http://www.badc.ac.uk), were used to derive daily mean and maximum temperature, daily mean and daily maximum 8-hour running mean ozone concentrations for the period May-September 1993-2003 for the 15 conurbations in England and Wales illustrated in Figure 1. These conurbations were the focus of ozone and heat exposure studies. Datasets of higher resolution hourly temperature (from BADC) and ozone (Air Quality Archive) from a greater number of monitoring sites, both urban and rural, were also obtained for UK-wide evaluation of the climate-chemistry model. A particular focus was the August 2003 heatwave period.

### Chemistry-climate model

Model representation of the physical and chemical state of the atmosphere at fine-scale resolution has been achieved through coupling of a numerical weather prediction model to a chemistry transport model. The domain used by both models covers the British Isles and parts of France, Denmark, Holland and Belgium. The two models operate at a horizontal resolution of 5 km × 5 km with ~20 vertical levels that extend from the surface up to 100 hPa (~16 km). The surface layer has depth 90 m. These regional-scale models are constrained by global meteorological and chemical boundary and initial conditions.

The chemistry transport model is EMEP4UK [17]. The chemical scheme is identical to the extensively validated EMEP Unified Model [18] (Europe domain at 50 km × 50 km) which is widely used for air quality studies that inform EU policymakers. EMEP4UK operates at a much higher resolution needed for epidemiological studies. The chemical mechanism is based on the photochemistry of ozone, whereby ozone is produced by the oxidation of carbon monoxide (CO), methane (CH_4_) and non-methane hydrocarbons (NMHCs) in the presence of nitrogen oxides (NO_x_). Anthropogenic emissions of NO_x_, CO, NMHC, and particles PM_2.5_, PM_CO _(coarse particulate matter) are derived from the UK's National Atmospheric Emissions Inventories. Biogenic temperature-sensitive emissions of isoprene and monoterpenes (NMHCs) are also included.

We use a nesting approach in order to perform 5 km by 5 km resolution simulations. First, we use the EMEP Unified Model to simulate chemical transport across a much larger European domain at 50 km × 50 km resolution. The model used climatologically derived ozone boundary and initial conditions. These simulations provide the chemical initial conditions and boundary conditions for the EMEP4UK 5 km by 5 km resolution model simulations. The EMEP Unified model and EMEP4UK are both driven by meteorology from the Weather Research Forecast (WRF) model [19]. WRF was also applied using a nesting approach whereby simulations were first performed at a 50 km by 50 km resolution for the larger European domain and these were used as boundary and initial conditions for further WRF simulations over the smaller UK domain at 5 km by 5 km resolution. These simulations provided the meteorological data to drive the EMEP European and EMEP4UK models at the required horizontal and vertical resolution. The re-analyses data from the US National Center for Environmental Prediction (NCEP)/National Center for Atmospheric Research (NCAR) Global Forecast System (GFS) were used as boundary conditions (6-hourly data assimilation) for the WRF European large-scale simulation.

Coupled WRF/EMEP4UK simulations have been performed for 2003, 2005 and 2006.

### Ozone- and heat-related mortality burdens

Estimates of ozone- and heat-mortality relationships were based on time series regressions of daily mortality data, for the period May-September 1993-2003 for the 15 English and Welsh conurbations, reported elsewhere [20]. In this regression model, mortality is the independent variable and ozone and temperature are dependent variables along with other confounding variables. To estimate the ozone-mortality relationship independent of temperature we used two-day means of daily maximum 8-hour ozone as the primary predictor in the regression model, controlling for temperature (represented as natural cubic splines), PM_10_, as well as day of week, and public holidays. Heat effects were obtained from this same model, comparing the adjusted mortality rate at the 97.5^th ^percentile of summer temperatures compared to that at the 75^th ^percentile. This analysis was also performed at the regional-scale (Table 1), based on regional data of England and Wales, which provides statistically more precise estimates of the heat-mortality relationship for health burden estimation (see below).

**Table 1:** Preliminary estimates of ozone- and heat-attributable deaths in England and Wales for combined years 2003, 2005 and 2006 using observed ozone heat-mortality relationships from Pattenden *et al.*[20] combined with simulated ozone concentrations and temperature from EMEP4UK.

	***Total deaths***	***Number and percentage (95% CI) of deaths *attributable to:**			
		***ozone***		***Heat***	
		***Number***	***percent***	***Number***	***percent***
**All**	596914	6798	1.1 (0.5 - 1.7)	5531	0.9 (0.8 - 1.1)
					
**By Region:**					
North East	31890	318	1.0 (0.5 - 1.5)	42	0.1 (-0.0- 0.3)
North West	83766	817	1.0 (0.5 - 1.5)	1068	1.3 (1.1 - 1.5)
Yorks & Hum	58502	559	1.0 (0.4 - 1.4)	352	0.6 (0.4 - 0.8)
E Midland	49183	531	1.1 (0.5 - 1.6)	420	0.9 (0.7 - 1.0)
W Midland	61324	668	1.1 (0.5 - 1.6)	591	1.0 (0.8 - 1.1)
East	61166	735	1.2 (0.6 - 1.8)	316	0.5 (0.4 - 0.6)
London	62277	756	1.2 (0.6 - 1.8)	1202	1.9 (1.8 - 2.1)
S East	89979	1199	1.3 (0.6 - 2.0)	688	0.8 (0.7 - 0.9)
S West	61663	785	1.3 (0.6 - 1.9)	519	0.8 (0.7 - 1.0)
Wales	37164	429	1.2 (0.5 - 1.7)	333	0.9 (0.7 - 1.1)
					
**By Population density:**					
Urban	394152	4492	1.1 (0.5 - 1.7)	4383	1.1 (0.9 - 1.3)
Other	202762	2306	1.1 (0.5 - 1.7)	1149	0.6 (0.4 - 0.7)
					
**By Month:**					
May	126148	1912	1.5 (0.7 - 2.3)	79	0.1 (0.0 - 0.1)
June	117910	1478	1.3 (0.6 - 1.9)	683	0.6 (0.4 - 0.7)
July	119909	1311	1.1 (0.5 - 1.6)	2717	2.3 (1.9 - 2.7)
August	119056	1142	1.0 (0.5 - 1.4)	1963	1.6 (1.4 - 1.9)
September	113891	956	0.8 (0.4 - 1.3)	89	0.1 (0.1 - 0.1)
					
**By Temperature:**					
<92nd centile	481556	4770	1.0 (0.5 - 1.5)	0	0.0 (0.0 - 0.0)
92nd-99th centile	92109	1516	1.6 (0.8 - 2.5)	2337	2.5 (1.9 - 3.2)
> = 99th centile	19168	512	2.7 (1.3 - 4.0)	3195	16.7 (14.9- 18.5)

Burdens of disease or health/mortality burdens are defined here as the number of deaths that can be attributed to ozone or heat exposure. The regional ozone-heat-mortality relationships described above were combined with the outputs of surface ozone and temperature from EMPEK4UK climate-chemistry simulations for years 2003, 2005 and 2006 and data on the counts of deaths from all causes from the Office of National Statistics (ONS) for the same period. The data were stratified by region, population density, month and temperature band (< 92^nd ^percentile, 92^nd^-99^th ^percentile, >99^th ^percentile) and were used to quantify ozone-heat related mortality burdens across England and Wales.

### Future emission scenarios

To perform WRF/EMEP4UK simulations of ozone concentrations under future emissions scenarios we will apply scaling factors to anthropogenic emissions data currently used in EMEP4UK (as above) based on three future emission scenarios (optimistic, pessimistic and current legislation) and use compatible initial and boundary chemical conditions from existing global Chemistry transport model simulations performed with these three same future emission scenarios [21].

## Results and discussion

### Ozone and temperature extremes

**Figure 2:**
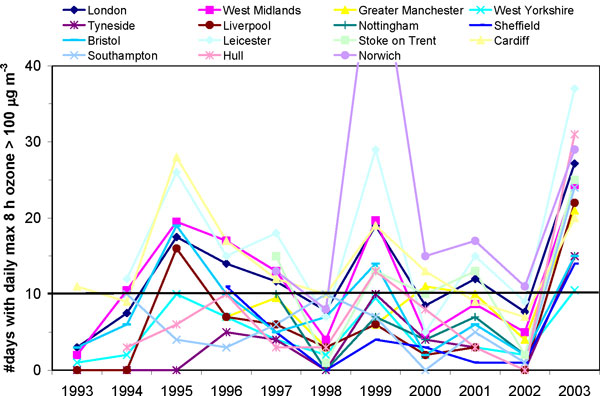
**Number of days per year when daily maximum 8 hour running mean ozone exceeded 100 μg m^-3 ^in each conurbation**. The current UK ozone objective for protection of human health is breached if there are more than 10 such days per annum (marked by the solid black line). Data are included only when ozone monitor data capture is sufficient. More than one monitor contributes to the data series for London, West Midlands, Greater Manchester and West Yorkshire.

The averages of the 1993-2003 May-September daily maximum 8-hour ozone concentrations across the fourteen largest English and Welsh conurbations in Figure 1 ranged between 52 μg m^-3 ^(26 ppb) and 65 μg m^-3 ^(32.5 ppb) with standard deviation values between 19-28 μg m^-3^. In Norwich, which was included for spatial representativeness as a 15^th ^conurbation, and may be regarded as the most rural-like location in these analyses, average daily maximum 8-hour ozone concentration was higher at 73 (± 28) μg m^-3 ^(37 ± 14 ppb). The current UK air quality objective for daily maximum 8-hour ozone (that was to be achieved by December 2005 and maintained thereafter) is 100 μg m^-3 ^(50 ppb), not to be exceeded more than ten days a year http://www.airquality.co.uk/standards.php. Figure 2 shows the number of days per year on which daily maximum 8 hour ozone exceeded 100 μg m^-3 ^for each of the 15 conurbations for the period analysed in this study. It can be seen that these urban areas have regularly had many days with 8 hour ozone concentrations greater than 100 μg m^-3^. In 2003, all 15 conurbations were in breach of the current ozone air quality objective, with more than 10 days of exceedences of this value. Populations in rural areas are generally exposed to higher ozone concentrations than neighbouring urban populations [22]. It is therefore of concern that the current evidence indicates that exposure of the UK population to ozone is likely to increase, rather than decrease, in the coming decades [22].

Average daily-mean temperatures for the 15 conurbations ranged between 14.0-16.4°C, whilst the threshold temperatures for designation as a "hot day" within each conurbation, defined as the 95^th ^percentile of 2-day mean temperature over the whole period 1993-2003, ranged between 17.6-19.8°C [20]. In Greater London (the largest conurbation) May-Sept mean daily maximum 8-hour ozone concentration was 60 μg m^-3^, mean daily temperature was 16.2°C, and the temperature threshold for "hot day" designation was 19.8°C [20]. The correlation coefficients between daily maximum 8-hour ozone and daily mean temperature for this May-Sept period were 0.39 for Greater London and 0.25 for the pooled dataset of all conurbations [20] indicating some evidence for a relationship between the two parameters.

**Figure 3:**
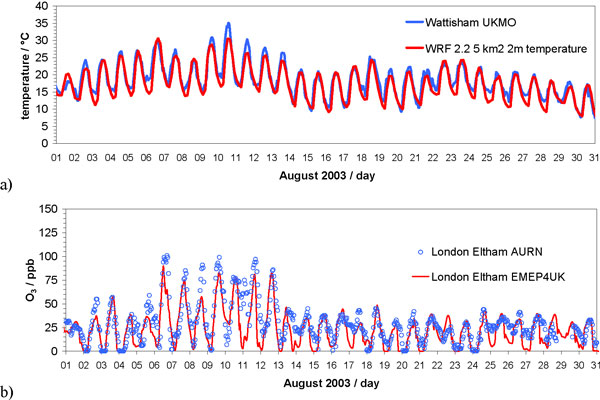
**For August 2003: (Upper plot) time series of hourly surface temperature measured at the Wattisham UK Met Office weather station (blue) and simulated by the WRF model for the surrounding 5 × 5 km^2 ^grid square (red); (Lower plot) time series of hourly WRF-EMEP4UK modelled (red) and measured (blue) surface ozone concentrations at London Eltham**. Units are ppb (1 ppb = 2 μg/m^3^).

Figure 3 shows example observed and modelled hourly surface temperatures and ozone concentrations in SE England for August 2003, which encompasses the Europe-wide heatwave period of 4^th^-12^th ^August. Daily maximum temperatures throughout this period exceeded 25°C, and exceeded 36°C on August 10^th ^2003, in Greater London and the surrounding area, e.g. as shown for Wattisham in Suffolk. The WRF model results clearly capture this heatwave episode (although it underestimates peak temperatures on several days) and the hourly results show good agreement with the observations during August 2003 (*r *= 0.9) [17]. The enhancement of surface ozone concentrations on hot sunny days during this period is clearly evident (Figure 3). Surface ozone concentrations rose substantially during this period with daily maximum concentrations at the urban background site of London Eltham reaching > 200 μg m^-3 ^(100 ppb) between 6^th ^and 13^th ^August 2003. As discussed above, this heatwave period contributed to breaches of the current UK ozone air quality objective at this site and all population centres in this study.

**Figure 4:**
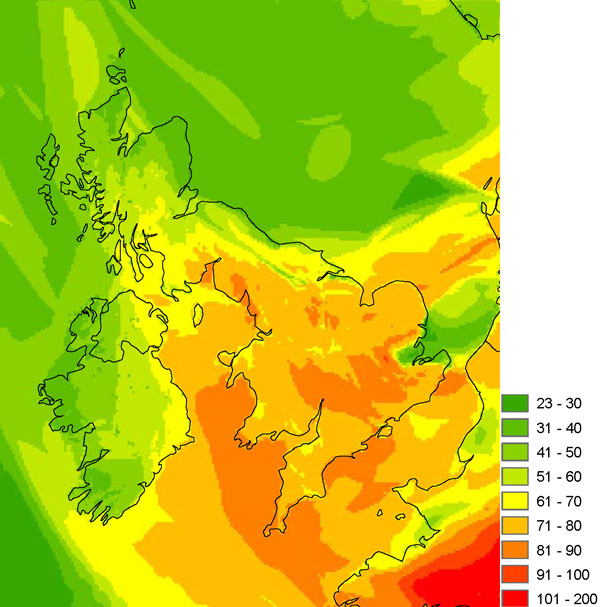
**EMEP4UK modelled daily-maximum surface  ozone concentrations for the 9^th ^August 2003 **[17]. Units are ppb (1 ppb = 2 μg/m^3^).

As shown in Figure 3, The EMEP4UK model is also able to capture the observed ozone concentrations during the heatwave (*r *= 0.77). Model-simulated surface ozone compared very well in general with monitor data from the UK Air Quality Archive. (For example, for the whole of 2003, across 34 urban and rural sites in England and Wales, the mean *r*-value was 0.80, and the mean RMSE was 14 μg m^-3^). However there was day-to-day variability in surface ozone concentrations during the heatwave period (Figure 3). Clearly other factors, besides the elevated temperatures and sunshine hours that give rise to enhanced photochemical production, have a role to play in determining surface ozone concentrations for a given location. The spatial pattern of daily maximum surface ozone concentrations across the UK on the 9^th ^August 2003 was strongly influenced by meteorological conditions that control transport of emission plumes and allow import of ozone formed over the European continent to the UK (Figure 4). During the heatwave period, local meteorological conditions and emissions as well as ozone import were found to be important in determining day-to-day variability in ozone concentrations. Through a series of EMEP4UK sensitivity experiments, the relative contributions of meteorological and chemical factors that influenced ozone concentrations on different days during the August 2003 heatwave were identified [17]. On several days background ozone produced on the European continent and imported into the UK was the main source of elevated surface ozone concentrations at a single site in the UK [17]. This illustrates the importance of simulating the large-scale chemical transport as well as local-scale emissions and chemical processes in order to determine surface local ozone concentrations accurately.

### Ozone and heat-related mortality burdens

Preliminary results of ozone and heat related mortality burdens for 2003, 2005 and 2006 combined are shown in Table 1. Overall, the number of deaths attributable to ozone appears slightly greater than that attributable to heat. Heat deaths show appreciable regional variation in terms of both the *number *and *proportion *of deaths attributable to heat, while the burden of ozone deaths was less varied in proportional terms. Proportionally more heat-deaths occur in urban settings, but the same is not true for ozone-related deaths. Ozone deaths appear to be greatest in the early warm-season (May, June) while heat deaths were greater in July and August, reflecting the warmer temperatures. Proportionally more ozone deaths occurred in periods of very high temperature.

The model simulations of simulated ozone concentrations for 2030 under future emissions scenarios will be used to calculate future health burdens and provide uncertainty estimates based on the choice of emission scenario. The three emission scenarios span a range of possible futures (described in detail in [16]) from:

a) assuming current legislation (CLE) and air quality standards apply in the future

b) calculating maximum feasible reductions (MFR) in the future (optimistic)

c) using the SRES A2 scenario which assumes regional economic growth, slower technological growth but rapidly continuous global population growth (pessimistic)

The first two of these emission scenarios were constructed at the International Institute for applied systems Analysis (IIASA), and the third of these by the Intergovernmental Panel on Climate change (IPCC) in its Special Report on Emissions Scenarios (SRES) [23]. Results from an inter-comparison of 26 global atmospheric chemistry models driven with these three future emissions scenarios show that global tropospheric ozone burden decreases by 5% and increases by 6% and 15% in the MFR, CLE and SRES A2 scenarios respectively [21]. The choice of scenario will undoubtedly affect surface ozone concentrations over the UK, the risk of exceedences of current UK air Quality objective standards and consequently the ozone-related health burdens [22].

## Conclusion

A new coupled climate-chemistry model is able to reproduce observed temperature and ozone trends in the UK, including photochemical ozone episodes and heatwaves which often occur in tandem. By combining climate-chemistry model simulations of surface temperature and ozone, with epidemiological evidence on ozone-heat-mortality relationships derived from observed data we estimate present-day and, subsequently, future health burdens across the UK for emission scenarios that span a range of possible futures (current legislation, optimistic, pessimistic). Given the predictions of increasing temperatures, future air quality legislation may need to consider the risk of increased incidences of high ozone concentrations associated with future increases in heatwaves.

## Note

The peer review of this article can be found in Additional file1

## Competing interests

The authors declare that they have no competing interests.

## Authors' contributions

All LSHTM authors contributed to the design of the model study. SP and BA constructed the epidemiological and health burden models. All Edinburgh authors contributed to the design and planning of the CTM simulations. MV developed the coupled model set-up and ran the model. All authors discussed future plans for the project.

## Supplementary Material

Additional file 1 Peer review. Click here for file
