# Supplementary material for: Current and future climate- and air pollution-mediated impacts on human health
Source: Environ Health. 2009 Dec 21;8(Suppl 1):S8. doi: 10.1186/1476-069X-8-S1-S8 (PMC2796504; doi:10.1186/1476-069X-8-S1-S8)
Supplement: Additional file 1 — Peer review. [file 1476-069X-8-S1-S8-S1.doc]

| Article ref no. |  |
| --- | --- |
| Title | **Impacts of Future Environmental Change on climate and air polluted mediated health** |
| Author(s) | Doherty et al |

**Recommendation: reconsider after essential revisions**

**Ref#1**

**General comments:**

I like the idea of linking statistical epidemiological models with simulation models and this paper shows the usefulness of this approach. The basic issue I have is with the lack detail presented which make it difficult to assess the robustness of the methodology used and the results obtained. I suspect this is due to the short paper format. However even given the limitations of the format I think the authors could make a better attempt to explain their methods and the significance of their results.

**Major compulsory revisions:**

Methods: there is a general tendency to assume knowledge rather than explain. This needs to be addressed.

The results section is poor. An explanation of the results and there significance would help, rather than just referencing other papers.

A comment in the conclusion concerning how these results might be used in the future would also help.

**Minor essential revisions:**

None

**Ref#2**

**General comments:**

This is an interesting paper but lacks details of the results: I guess that these are being published elsewhere. However, if the paper is to be acceptable for publication then there needs to be more substantive data included.

**Major compulsory revisions:**

The paper assumes prior knowledge of the methodology and needs to be presented in a clearer “reader-friendly” form for those not familiar with this topic.

**Minor essential revisions:**

None

**Ref#3**

**General comments:**

This paper is not acceptable in its current form. It lacks sufficient depth as the data is probably being published elsewhere.

# Responses to Referees

We have substantially revised the paper based on the reviewers’ comments and on your guidance as guest editor to produce a much improved project overview paper. We have re-written our methodology for a more diverse audience, provided more details and included new results (that are not published elsewhere) that outline our approach and linkages to calculate present-day UK health burdens, and most importantly discuss the relevance of our results within a policy context. Our specific responses are given below.

***Reviewers comments:***

*Methods:*

Ref #1:

Methods: there is a general tendency to assume knowledge rather than explain. This needs to be addressed.

Ref #2

The paper assumes prior knowledge of the methodology and needs to be presented in a clearer “reader-friendly” form for those not familiar with this topic.

***We have substantially revised the “methods” section to provide greater detail of our project approach and to improve readability for a wider interdisciplinary audience.***

*Results and Discussion*:

Ref #1

An explanation of the results and there significance would help, rather than just referencing other papers. A comment in the conclusion concerning how these results might be used in the future would also help.

## Ref #2

…lacks details of the results: I guess that these are being published elsewhere. However, if the paper is to be acceptable for publication then there needs to be more substantive data included.

Ref #3

It lacks sufficient depth as the data is probably being published elsewhere.

***We have re-written our results section. It now includes results not published elsewhere and discusses these within a policy context. These new results now include preliminary present-day health burdens. We have expanded our discussion of how our results will be used in the future to produce UK-wide present-day and future health burdens. Specifically, we have:***

1) ***written a new first section in the results entitled “Temperature and Ozone extremes.” This section brings together our analysis of environmental monitoring data for 15 UK conurbations and of measured and modelled temperature and ozone extremes during the 2003 heatwave. We discuss these results within the context of the UK air quality objective standards.***
2) ***Moved the text on “simulation of future scenarios” to the “methods” section. We have replaced this with a new results section entitled “Present-day and future health burdens” which discusses our rationale for calculating present day and future health burdens and our expected outcomes. This work represents our most recent findings and one of the core outcomes of our project. This replaces a section entitled “ozone-heat mortality relationships” that is submitted for publication elsewhere.***
